# Engineering the pH-Sensitivity of the Graphene and Carbon Nanotube Based Nanomedicines in Smart Cancer Therapy by Grafting Trimetyl Chitosan

**DOI:** 10.1007/s11095-020-02881-1

**Published:** 2020-08-03

**Authors:** Azadeh Khoshoei, Ebrahim Ghasemy, Fatemeh Poustchi, Mohammad-Ali Shahbazi, Reza Maleki

**Affiliations:** 1 Institute of Nano Science and Nano Technology, University of Kashan, Kashan, Iran; 2 Nanotechnology Department, School of New Technologies, Iran University of Science and Technology, Tehran, Iran; 3 Department of Nanotechnology, University of Guilan, Guilan, Iran; 4 Drug Research Program, Division of Pharmaceutical Chemistry and Technology, Faculty of Pharmacy, University of Helsinki, FI-00014 Helsinki, Finland; 5 Zanjan Pharmaceutical Nanotechnology Research Center (ZPNRC), Zanjan University of Medical Sciences, Zanjan, 45139-56184 Iran; 6 Department of Chemical Engineering, Shiraz University of Technology, Shiraz, Iran

**Keywords:** cancer therapy, graphene, molecular dynamic, nanomedicine, single walled carbon nanotube

## Abstract

**Purpose:**

The aim of this study was to introduce a smart and responsive drug carrier for Doxorubicin (DOX) and Paclitaxel (PAX) for desirable therapeutic application.

**Method:**

Loading and releasing of DOX and PAX from smart and pH-sensitive functionalized single-walled carbon nanotube (SWCNTs) and graphene carriers have been simulated by molecular dynamics. The influences of chitosan polymer on proposed carriers have been studied, and both carriers were functionalized with carboxyl groups to improve the loading and releasing properties of the drugs.

**Results:**

The results showed that DOX could be well adsorbed on both functionalized SWCNTs and graphene. In contrast, there was a weak electrostatic and Van der Waals interaction between both these drugs and carriers at cancerous tissues, which is highly favorable for cancer therapy. Adding trimethyl chitosan (TMC) polymer to carriers facilitated DOX release at acidic tissues. Furthermore, at blood pH, the PAX loaded on the functionalized SWCNTs carrier represented the highest dispersion of the drug while the DOX-graphene showed the highest concentration of the drug at a point. In addition, the mean-square displacement (MSD) results of PAX-graphene indicated that the PAX could be adsorbed quickly and be released slowly. Finally, functionalized graphene-TMC-PAX is a smart drug system with responsive behavior and controllable drug release, which are essential in cancer therapy.

**Conclusion:**

Simultaneous application of the carboxyl group and TMC can optimize the pH sensitivity of the SWCNTs and graphene to prepare a novel and smart drug carrier for cancer therapy.

## Introduction

Many new cases of cancers are being reported by the World Health Organization annually and many different efforts have been made to cure or prevent the disease (1). However, for most patients, the local invasion and distant metastasis are often available and only a few patients have the chance to receive early diagnosis and treatment (2). As chemotherapy drugs target all rapidly dividing cells, including both healthy and cancerous cells, they can cause severe side effects too. Hence, considering the newly developed methods to detect cancer at early stages and targeted drug delivery to cancerous cells without impacting healthy cells would be desirable. Recently, the biomedical field using nanomaterials has gained wide applications in early disease diagnosis and effective drug delivery into the purposed cells (3–6). In this regard, Carbon Nanotubes (CNTs) have been extensively for various applications due to their tunability characteristic, which can also cross the cell membranes (7–11). Over the last few decades, numerous methods of modifying CNTs have been used in cellular therapy, drug delivery, and sensors for detecting specific proteins and other biomolecules in serum. Functionalized carbon nanotubes (f-CNTs) have become the sites of interest (12,13) in recent researches. Graphene has also been reported as a promising material for various applications ranging from quantum physics, catalysis, and engineering of nanocomposites nanoelectronics to energy research and biomaterials (14–17). In a nanomedicine realm, graphene and its composites can be employed in different applications including a new generation of biosensors, nanocarriers for drug delivery, and probes for cell and biological imaging (15,18–20). During the last two decades, different nanomaterials of various shapes and chemical compositions, including metal and metal oxide nanoparticles, polymeric micelles, liposomes, dendrimers, and carbon nanotubes, have been studied as nano-carriers for drug delivery (21–24).

Doxorubicin (DOX) and paclitaxel (PAX) are two main anticancer drugs that can be used in cancer chemotherapy (25,26) to heal a wide range of cancers in the lung, breast, and ovaries (27,28). In recent researches, the coupling of the DOX and PAX has shown advantageous combinational therapy. The importance of performing theoretical studies before the experiments has become widespread, and they should be done to ensure the efficiency and reduce the side effects accompanied by various drug systems. It is clear that the properties of carbonaceous materials, such as single-walled carbon nanotubes (SWCNTs) can be improved and tuned through applying various functionalizing agents or dopants which has enabled the researchers to develop novel carbon nanostructures with optimized performance. Furthermore, the carbon nanomaterials can respond to specific conditions through which a smart material can be developed (29,30).

It is well established that simulations can be highly useful in developing novel and smart nanomedicine systems. In this regard, many efforts have been devoted to exploiting Molecular Dynamics (MD) (31–35), Density Functional Theory(DFT) (36), and Machine Learning (37–39) in discovering emerging and smart drug nanocarriers. Shariatinia and Mazloom-Jalali (40) perused the graphene containing chitosan as a carrier of anticancer ifosfamide drug through MD, in which, the N-doped graphene/chitosan was suggested as an effective drug carrier. Furthermore, Shang *et al*. (32) investigated the effect of Zn on the DOX adsorption capacity of hydroxyapatite. The MD simulations clarified that Zn-doping can enhance the DOX adsorption capacity of the hydroxyapatite. These studies showed that MD calculations could be performed to even propose smart nanocarbon based drug carriers, which are pH and temperature-sensitive and can provide precise loading and controllable release of the drugs to the targeted tissue (41). Herein, a MD study was conducted to develop smart and responsive drug delivery systems. In this regard and to induce a smart behavior, trimethyl chitosan (TMC) and carboxyl groups have been used as a grafting and functionalizing agents, respectively, for the co-adsorption and targeted delivery of DOX and PAX by SWCNTs and graphene to the cancerous cells.

## Materials and Methods

### Force Fields

The precision and validity of simulation results in MD can be achieved by using Force Fields. Changing the distances of inter-particles will cause the Interactive energies (potential energy) changes. The potential function is presented by Eq. (1). Moreover, the force function of each *i* atom in an N-atom system can be obtained from Eq. (2) which is extracted from the potential function. These equations are solved at the same time. Besides, the force is specified to time and the atomic position by using Eq. (3) (42,43). Simple potentials such as the hard-sphere potential can be employed for primary molecular simulation. It is assumed that the particles with a constant velocity move in straight lines. As the distance of a sphere equals to the sum of their radii, relatively elastic collisions are going to happen.

According to the principles of conservation of linear motion size, a new velocity will be calculated. By using the hard-sphere model, proper results can be obtained, although it is not ideal in atomic or molecular system simulations. As interatomic or intermolecular distances vary, based on the Van der Waals potential, their forces change. Eq. (4) represents the Van der Waals potential, where *σ* indicates the potential well depth, and *q* denotes the distance at which the potential becomes zero. The distance between two atoms is shown by *r*, and their interatomic potential is illustrated by *V* (44):

1 $$U=u(r)$$

2 $$Fi=\frac{dv}{dri}$$

3 $$mi\frac{d^2 ri}{dt^2}= Fi,i=1,\dots, N$$

4

Equations (5) and (6) can be utilized to calculate the drug diffusion coefficient. Mean-square displacement (MSD) was considered to determine the drug diffusion coefficient; in which the coordinates of the atoms are also given as *r* while *t* implies the time. By using Einstein’s relation (Eq. (6)), the diffusion coefficient can be calculated for the three-dimensional system, after calculating MSD (45,46):

5 $$MSDS=\left\langle {\left[r(t)-r(0)\right]}^2\right\rangle =\frac{1}{t}\sum \limits_{t=0}^t\ {\left[\mathrm{r}\left(\mathrm{t}\right)\hbox{--} \mathrm{r}(0)\right]}^2$$

6 $$\underset{t\to \infty }{D=\frac{1}{6}\lim}\frac{MSD}{t}$$

In this work, GROMACS open source software was used in the drug simulation.

### System Preparation

In this paper, we will explore the effects of functionalizing the SWCNTs and graphene and grafting TMC polymer in co-adsorption and controllable co-release of two main anti-cancer drugs; namely DOX and PAX. In this simulation, SWCNTs and graphene surface was functionalized with carboxyl groups to promote the drug loading (at neutral pH, equivalent to blood condition) on the carrier and controlled releasing (at acidic pH, equivalent to the cancerous tissues) the drug. There are two major purposes in functionalizing the surface of SWCNTs and graphene with carboxyl groups. First of all, a strong electrostatic interaction between carriers and the drugs will be achieved by functionalizing the surface using the carboxyl group. There are amine groups with positive surface charges in the DOX structure, which lead to a strong electrostatic interaction between carriers and the drugs when they are exposed to carboxyl groups with negative surface charges. Accordingly, the drug can be adsorbed on the carrier strongly. At acidic pH, the carboxyl groups have no surface charge, and the electrostatic interaction will be decreased. In fact, blood has neutral pH while the cancer tissues have acidic pH, so it seems to be necessary to have a carrier with a strong electrostatic interaction at blood pH to have easy adsorption of the drug at neutral pH; on the contrary, to ease the controllable release of the drug at acidic pH (cancerous tissues), the carrier should not possess electrostatic interactions at pH values of about 5.5. The second main role of the carboxyl group belongs to their polar property that can modify the hydrophilicity of the carrier to prevent drug accumulation in the blood. Furthermore, TMC was used to improve the hydrophilicity and solubility of the carbon-based carriers. Also, TMC can facilitate the release of the drug in cancerous tissues. SWCNTs and graphene surface was covered with carboxyl groups in both protonated and deprotonated modes. In doing so, zero surface charge was regarded while using the optimized potentials for liquid simulations all atoms (OPLSaa) force field. Charge and other relevant parameters of the nanostructure functional groups were specified based on similar structures available in the OPLSaa force field. For determination of non-bonded interactions (i.e., electrostatic and Van der Waals), Lenard-Jones and Columbian potential models were used.

**Fig. 1:**
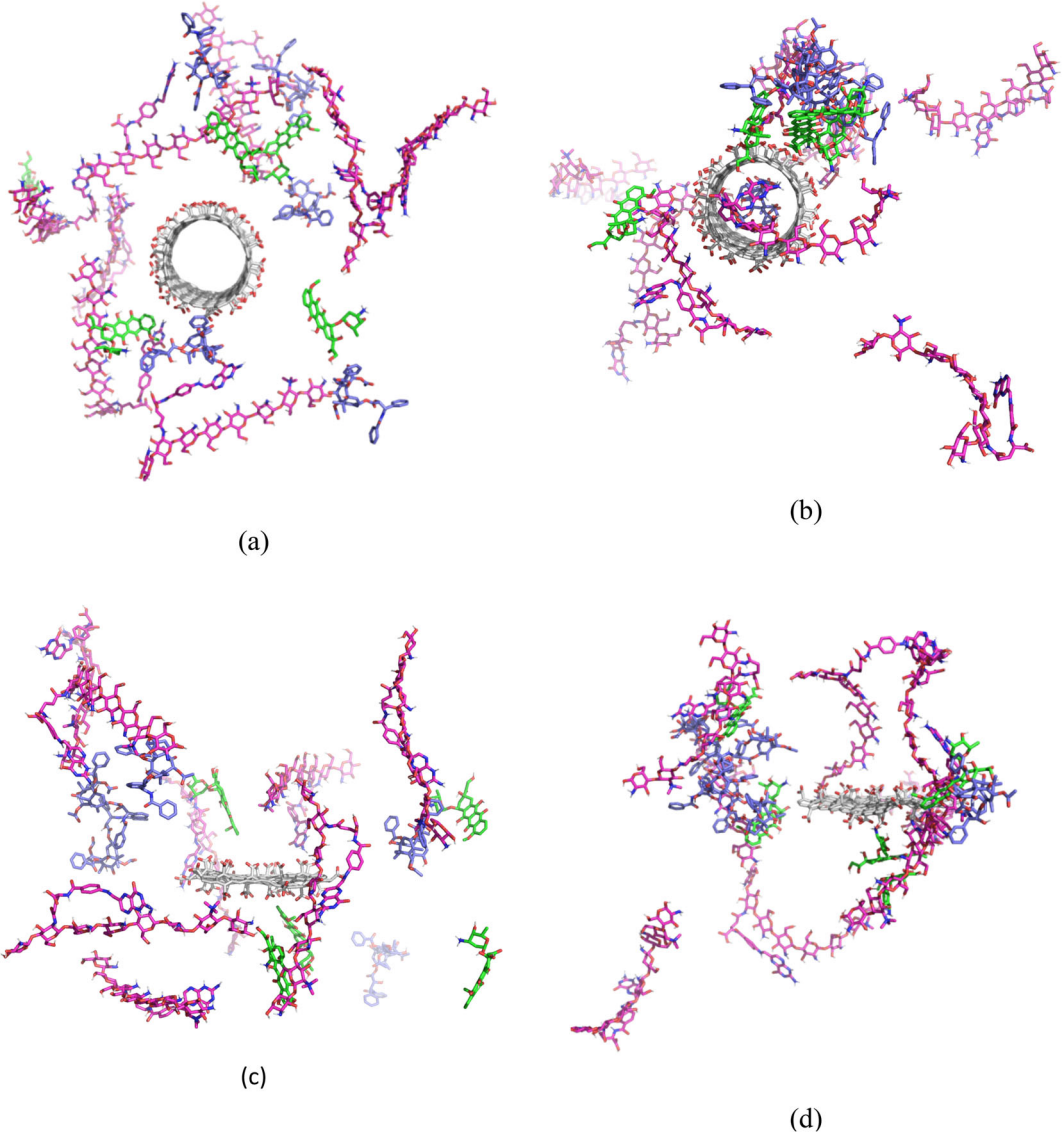
Structure of the SWCNT based drug nanosystem (**a**) before and (**b**) after simulation at the neutral pH. Structure of graphene-based drug nanosystem (**c**) before and (**d**) after simulation at the neutral pH.

All molecules were placed in the box, and the tip3p water model was employed as a solvent to achieve molecules parameters. To minimize the energy of all simulation systems, 5000 steps were implemented. In the next stage, to elevate the temperature of the system from 0 to 310 K in 100 ps in constant volume, the Nose–Hoover algorithm was used. Then, the system was balanced at a constant pressure in 200 ps. For system pressure balance, the Parrinello–Rahman algorithm was employed. The MD simulation was done at a temperature of 37°C for 50 ns. The cut-off distance was considered to 1.2. By using particle mesh ewald (pme), the electrostatic calculation was conducted. The linear constraint solver algorithm was applied for maintaining the bond lengths; the calculations were accelerated by implementing the SHAKE algorithm to limit the bonds engaged in the hydrogen atom. Composition of the drug nanosystems considered in the simulations along with the structures after performing the simulations (at neutral pH, adsorption) are shown in Fig. 1.

## Results and Discussion

### DOX–SWCNTs and DOX–Graphene Interactions

**Fig. 2:**
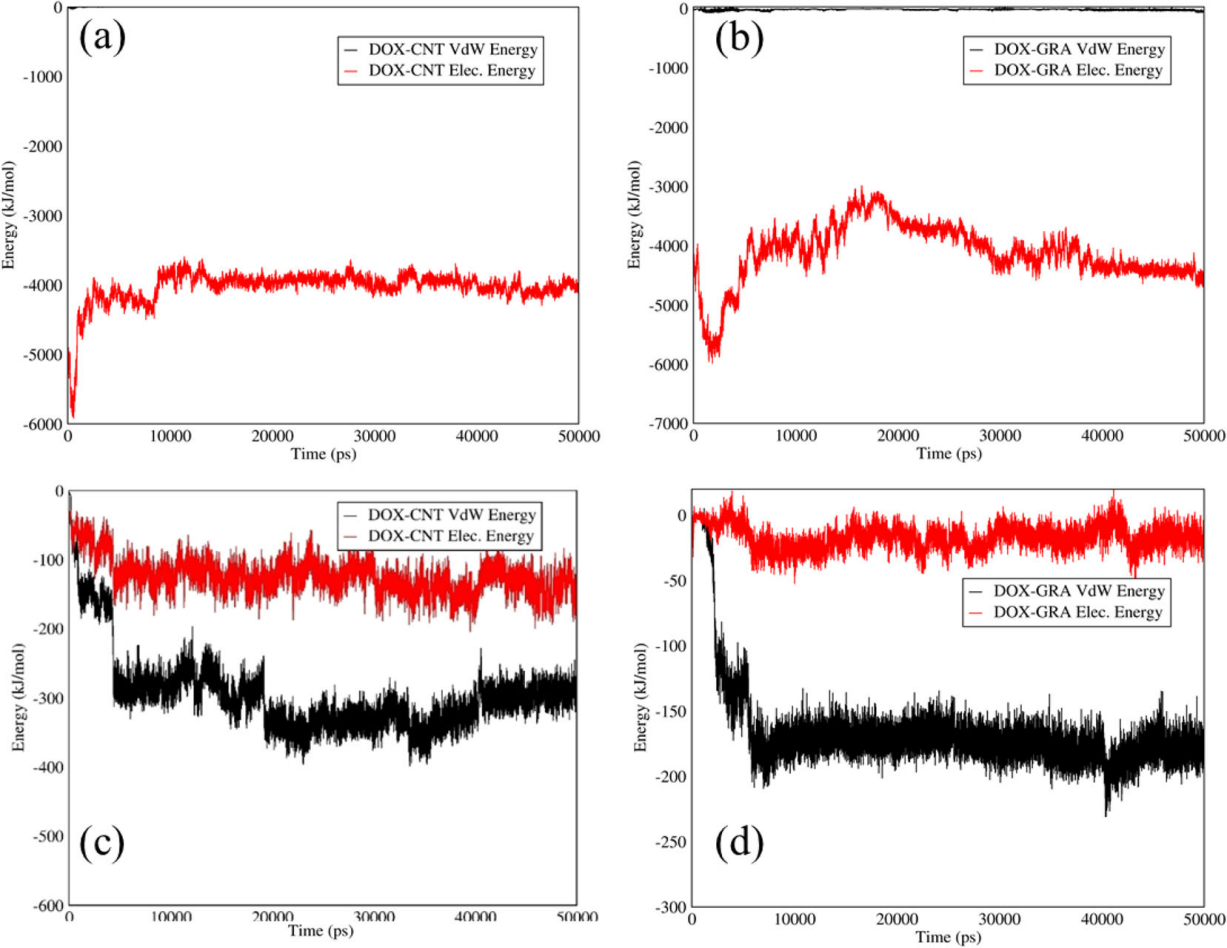
Electrostatic and Van der Waals energies of (**a**) DOX–SWCNTs and (**b**) DOX–graphene interactions *versus* time at neutral pH; and Electrostatic and Van der Waals energies of (**c**) DOX–SWCNTs and (d) DOX–graphene interactions *versus* time at acidic pH.

Van der Waals and electrostatic interactions between DOX-SWCNTs and DOX-graphene at the releasing and adsorption pH are indicated in Fig. 2. At neutral pH, according to Fig. 2(a) a strong electrostatic interaction around 4000 (kJ/mol) was observed between the DOX and the functionalized SWCNTs as a carrier. Indeed, the positive surface charge of the amine groups in the DOX structure was attracted by the negative surface charge of carboxyl groups in the functionalized SWCNTsʼ structure. Moreover, the DOX and functionalized graphene illustrated obvious adsorption at the neutral condition, which is related to the surface charge of functionalized graphene and amine groups of the DOX, as evident in Fig. 2(b). At acidic pH, carboxyl groups had no surface charge; therefore, both carriers indicated a weak interaction with the drug and a slow release, as shown in Fig. 2 (c,d). The reports of the study at neutral pH indicated a strong adsorption affinity by the carriers in which the carboxyl groups had negative surface charge, and DOX contained amine groups with positive surface charge.

### PAX–SWCNTs and PAX–Graphene Interactions

**Fig. 3:**
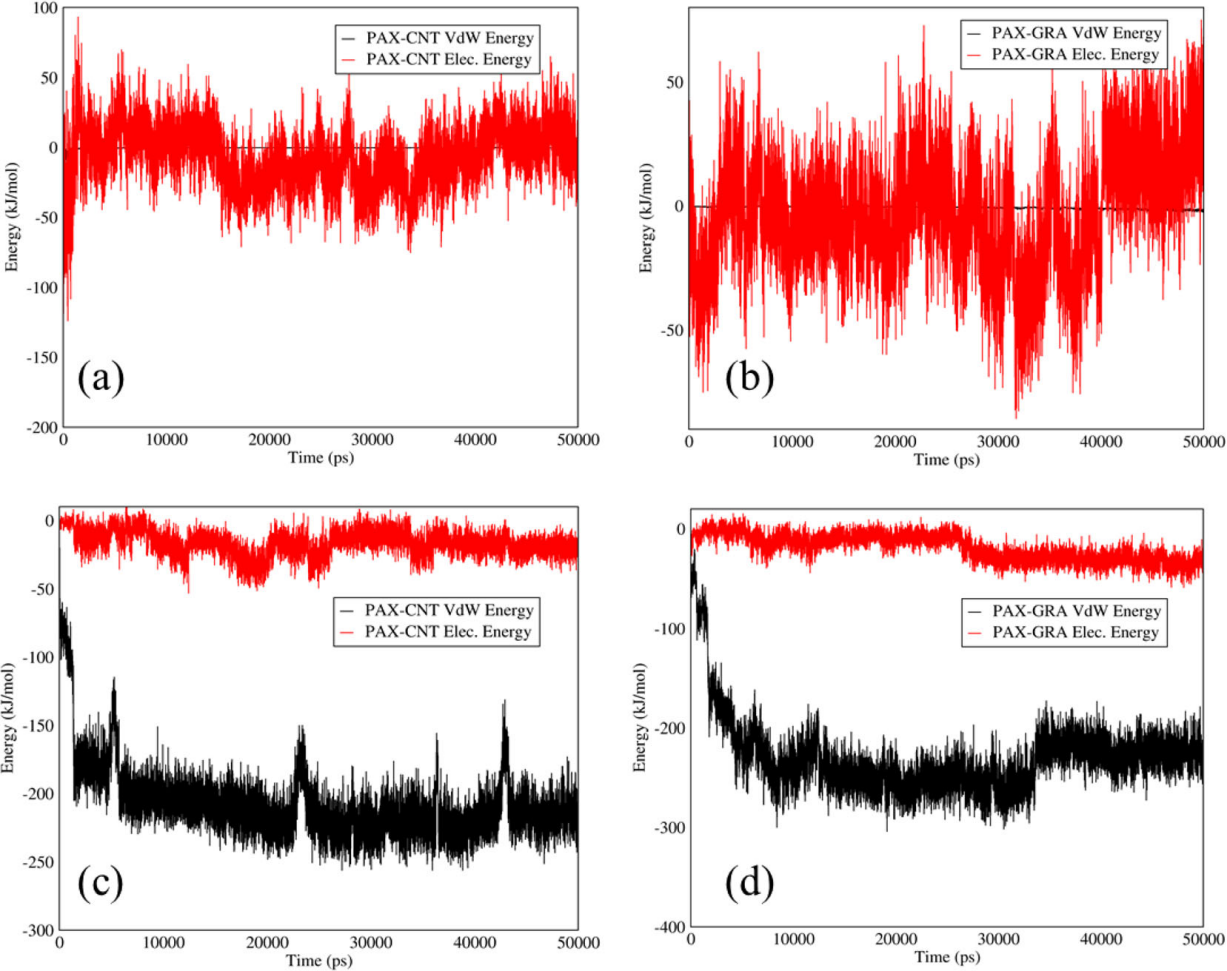
Electrostatic and Van der Waals energies of (**a**) PAX–SWCNTs and (**b**) PAX–graphene interactions *versus* time at neutral pH; electrostatic and Van der Waals energies of (**c**) PAX–SWCNTS and (**d**) PAX–graphene interactions *versus* time at acidic pH.

Figure 3 illustrates Van der Waals and electrostatic interactions between PAX-SWCNTS and PAX-graphene at releasing and adsorption pH. As it can be observed in Fig. 3, both drugs had a weak Van der Waals and electrostatic interactions at both acidic and neutral pH. In essence, PAX had no surface charge in its structure and in comparison with DOX that contained Amine groups with positive surface charge, PAX could not be adsorbed on the functionalized SWCNTs and graphene with carboxyl groups. According to Fig. 3 (c,d), PAX could be released slowly from SWCNTs and graphene carriers.

### DOX–TMC Interactions in DOX–TMC-SWCNTs and DOX–TMC-Graphene

**Fig. 4:**
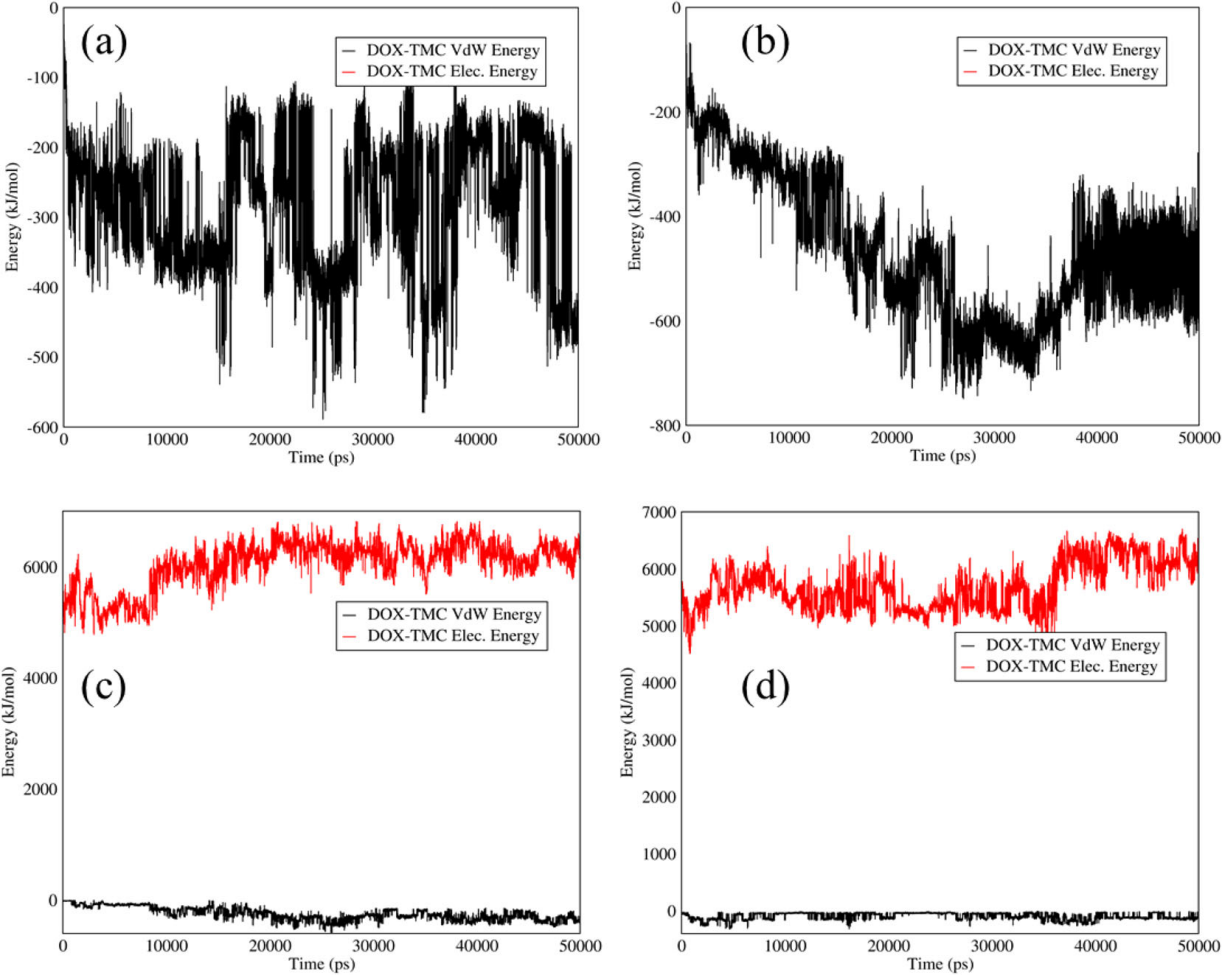
Electrostatic and Van der Waals energies of (**a**) DOX–TMC-SWCNTS and (**b**) DOX–TMC-graphene interactions *versus* time at neutral pH; Electrostatic and Van der Waals energies of (**c**) DOX–TMC-SWCNTs and (**d**) DOX–TMC-graphene interactions *versus* time at acidic pH.

It is proved that TMC can enhance the hydrophilicity and biocompatibility of different structures (47,48). To improve the hydrophilicity and biocompatibility of the carriers, TMC was added to the carriers. The electrostatic and Van der Waals interactions between DOX and TMC in the mixtures of DOX–TMC-SWCNTs and DOX–TMC-graphene at adsorption and releasing conditions are shown in Fig. 4. Based on Fig. 4 (a, b), there was a weak interaction between TMC and DOX in these systems at neutral pH. On the other hand, adding TMC to these carriers led to an increase in the drug release rate at acidic pH to approximately 5000 (kJ/mol), according to Fig. 4 (c,d), which is not appropriate in the drug delivery.

### PAX–TMC Interactions in PAX–TMC-SWCNTs and PAX–TMC-Graphene

**Fig. 5:**
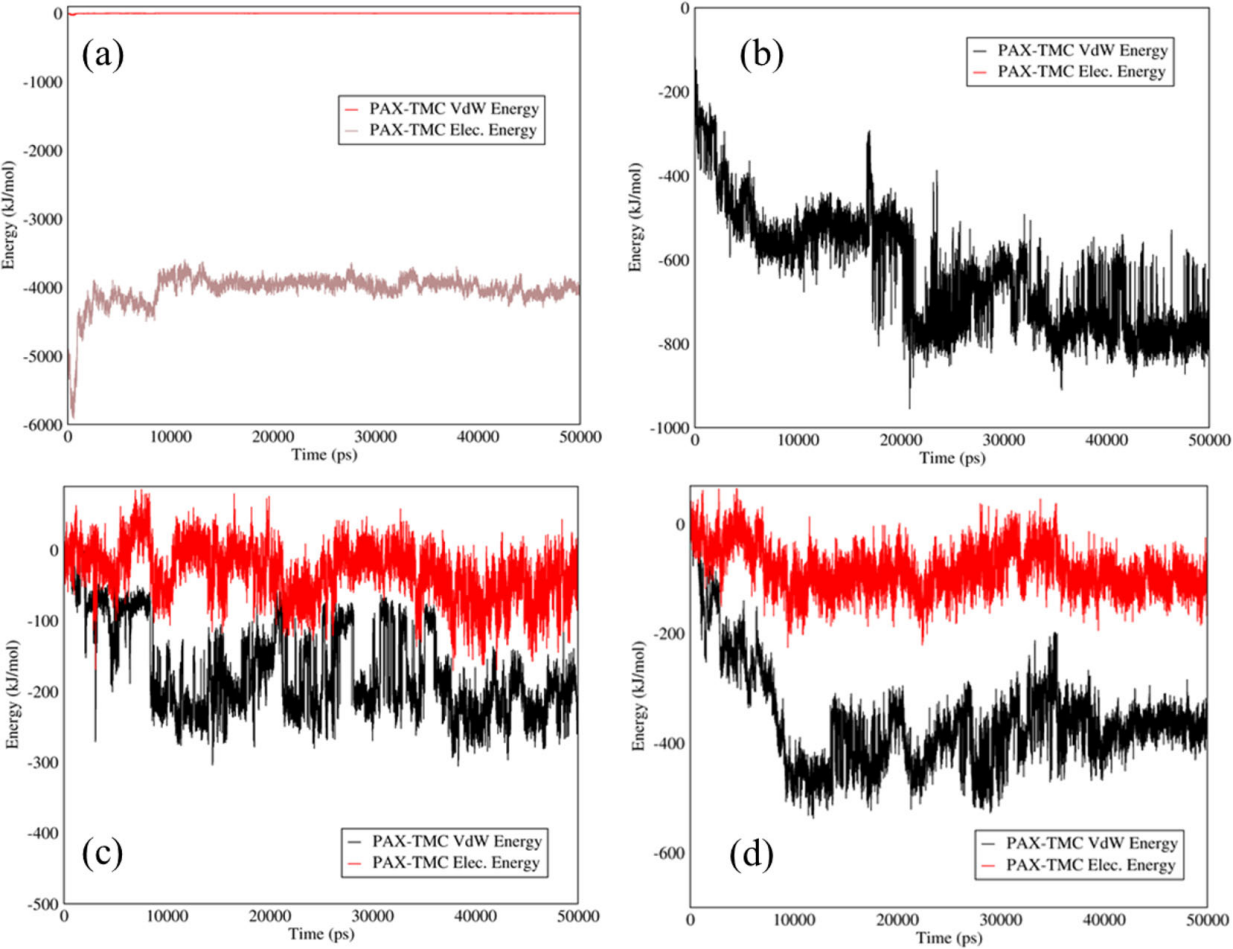
Electrostatic and Van der Waals energies of (**a**) PAX–TMC-SWCNTs and (**b**) PAX–TMC-graphene interactions *versus* time at neutral pH; Electrostatic and Van der Waals energies of (**c**) PAX–TMC-SWCNTs and (**d**) PAX–TMC-graphene interaction *versus* time at acidic pH.

The influences of adding TMC to the mixtures of PAX-SWCNTs and PAX-graphene are displayed in Fig. 5. An extraordinary augment in the electrostatic energy between PAX and TMC in the mixtures of PAX–TMC-SWCNTs and PAX–TMC-graphene can be observed in Fig. 5 (a,b) that attest to the fact that the addition of TMC can assist in the drug adsorption at neutral pH. Moreover, PAX could be released gradually, as can be observed in Fig. 5 (c,d) that verifies the appropriate release pattern of PAX from both mixtures.

### Hydrogen Bonds in DOX–SWCNTs and DOX–Graphene

**Fig. 6:**
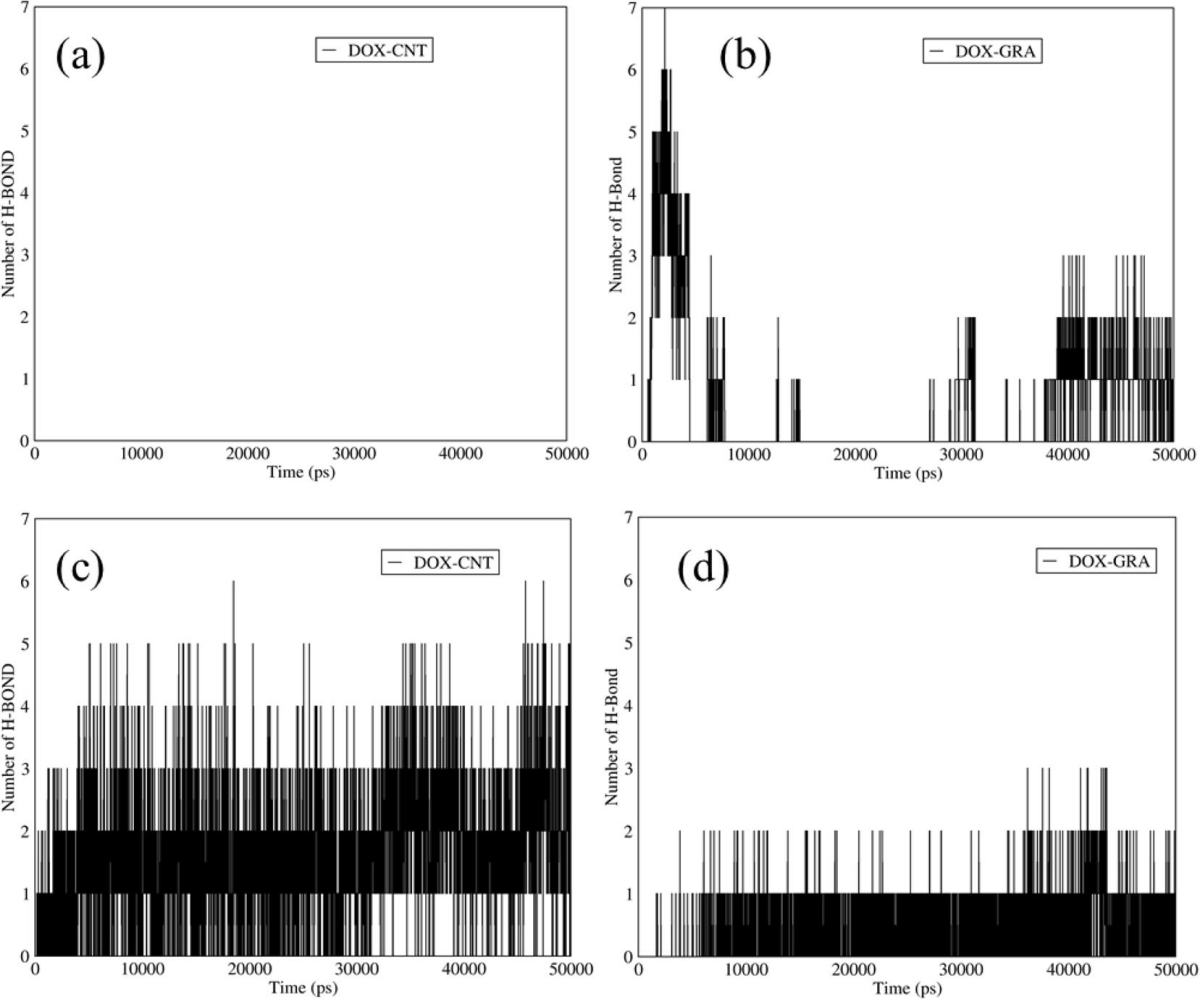
The number of hydrogen bonds between DOX and (**a**) SWCNTs and (**b**) graphene *versus* time at neutral pH; The number of hydrogen bonds between DOX and (**c**) SWCNTs and (**d**) graphene *versus* time at acidic pH.

Figure 6 shows the number of hydrogen bonds between the DOX and two different functionalized carriers at acidic and neutral pH. As it is indicated in Fig. 6 (a,b), the number of hydrogen bonds between the DOX and functionalized graphene was more than that of the functionalized SWCNTs at the adsorption condition. The main reason for the mentioned issue belongs to the spatial blockage of the drug and SWCNTs, which is referred to the electrostatic interaction between amine and carboxyl groups. There were a few hydrogen bonds between GO and DOX. Actually, graphene is a flat sheet in which the atoms can interact with the DOX atoms from the lower and upper surfaces of graphene, and it had a lower blockage spatial in comparison with SWCNTs, which has a cylindrical shape. Furthermore, both carriers were hydrophobic, and even adding carboxyl groups to the SWCNTs structure could not help to form the hydrogen bonds between SWCNTs and the drug. At acidic pH (Fig. 6 (c,d)), the number of hydrogen bonds in both carriers was increased because the spatial hindrance initiated from the carboxyl group of the functionalized carriers and amine group in DOX structure was declined. In fact, the carboxyl group had no surface charge at acidic pH, and there was a low electrostatic interaction between the DOX and these carriers, which led to a reduction of the spatial hindrance; thus, more hydrogen bonds could be observed at acidic pH.

### Hydrogen Bonds in PAX–SWCNTs and PAX–Graphene

**Fig. 7:**
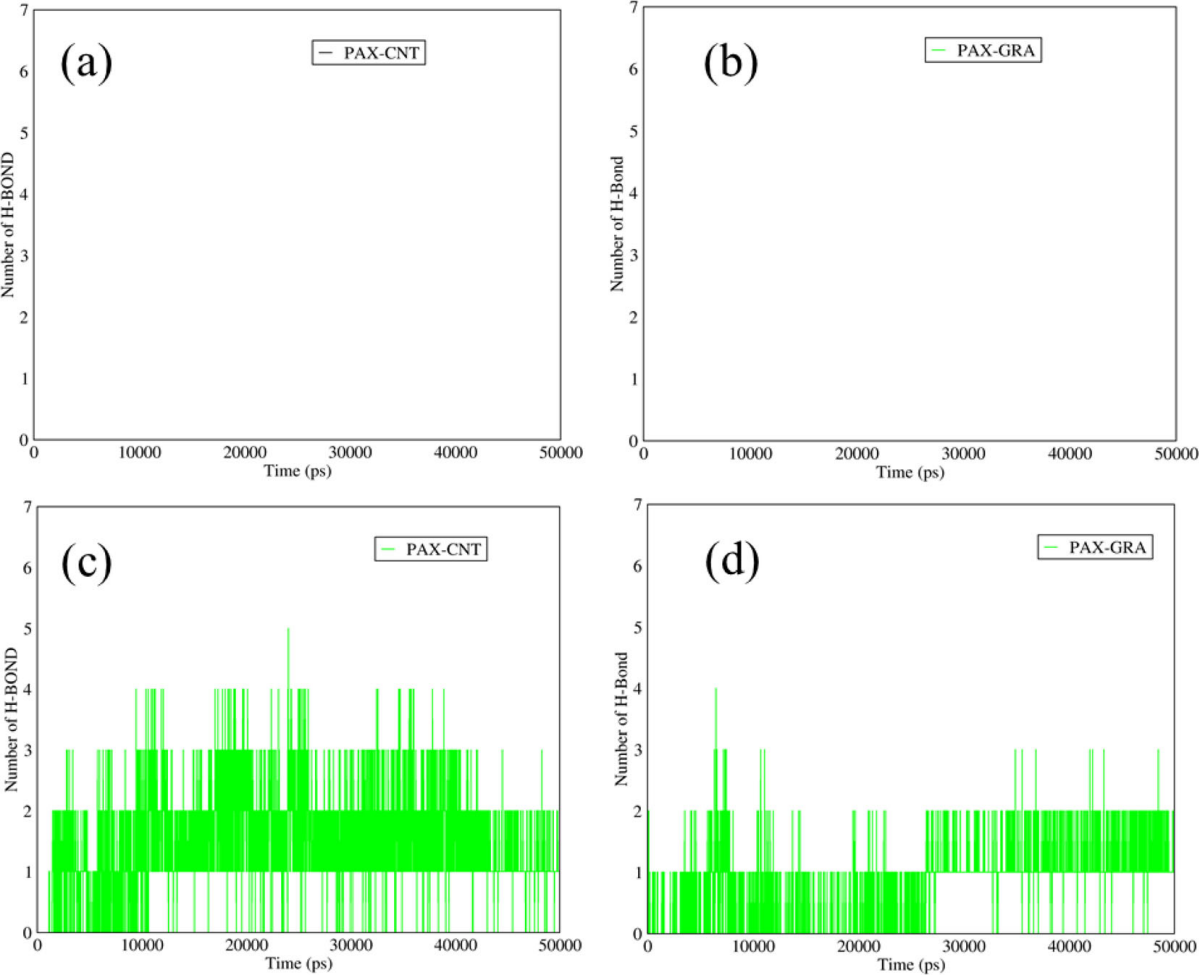
The number of hydrogen bonds between the DOX and (**a**) SWCNTs and (**b**) graphene *versus* time at neutral pH; The number of hydrogen bonds between the PAX and (**a**) SWCNTs and (**d**) graphene *versus* time at acidic pH.

The hydrogen bonds between the PAX and the proposed nanocarriers are illustrated in Fig. 7. According to Fig. 7 (a,b) at neutral pH, there were no hydrogen bonds between PAX and both carriers, which mentions the spatial blockage that was originated from the strong adsorption of DOX on these carriers. Indeed, at neutral pH, there was a significant electrostatic interaction the between amine groups of the DOX and carboxyl groups in functionalized structures of SWCNTs and graphene. Amine groups with positive surface charge were adsorbed considerably by the carboxyl groups in the structures of the carriers, which led to a remarkable spatial hindrance, so there were no hydrogen bonds at neutral pH. At acidic pH, the carboxyl groups of the functionalized SWCNTs and graphene had no surface charge, so the electrostatic interactions between the DOX and these functionalized carriers were declined. Therefore, approximately two hydrogen bonds could be seen between PAX and these carriers (Fig. 7 (c,d)).

### Hydrogen Bonds between DOX and TMC in DOX-TMC-SWCNTs and DOX-TMC-Graphene

**Fig. 8:**
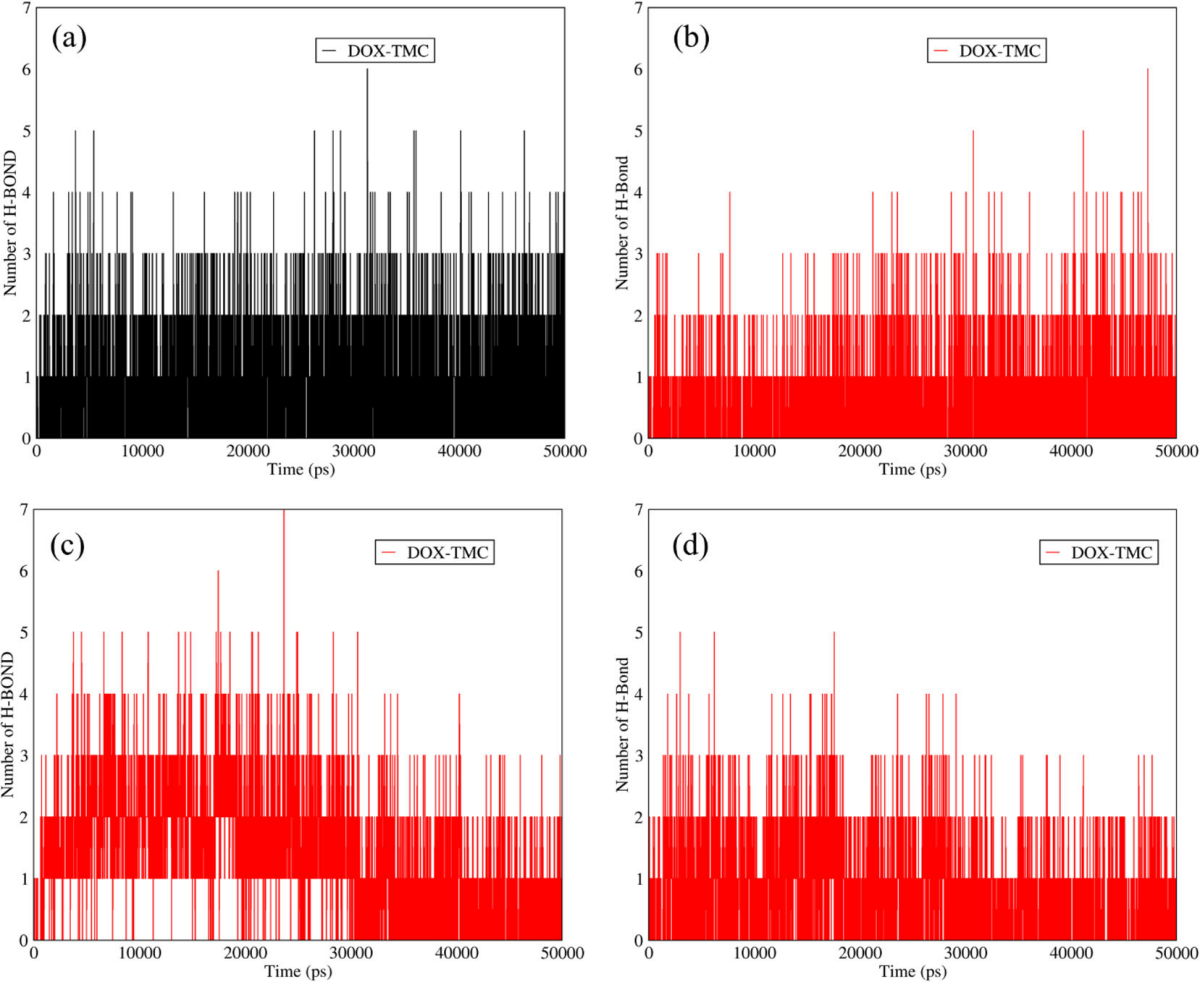
The number of hydrogen bonds between DOX and TMC in (**a**) DOX-TMC-SWCNTs and (**b**) DOX-TMC-graphene *versus* time at neutral pH; The number of hydrogen bonds between DOX and TMC in (**c**) DOX-TMC-SWCNTs and (**d**) DOX-TMC-graphene *versus* time at acidic pH.

The addition of the TMC to the structures of functionalized SWCNTs and graphene improved the hydrophilicity and biocompatibility of these carries. The presence of the TMC in the structures of the carriers resulted in the higher number of hydrogen bonds at adsorption condition between DOX and TMC (Fig. 8 (a,b)). Based on Fig. 8 (a,b), the average number of hydrogen bonds between the DOX and TMC was around two. Besides, at acidic conditions, there were almost two hydrogen bonds between the DOX and TMC for the mixtures of DOX-TMC-SWCNTs and DOX-TMC-graphene (Fig. 8 (c, d)). Accordingly, adding the TMC to the mixtures of these carriers, in addition to enhancing the hydrophilicity of these carries, could provide more hydrogen bonds of DOX with SWCNTs and graphene.

### Hydrogen Bonds between PAX and TMC in PAX-TMC-SWCNTS and PAX-TMC-Graphene

**Fig. 9:**
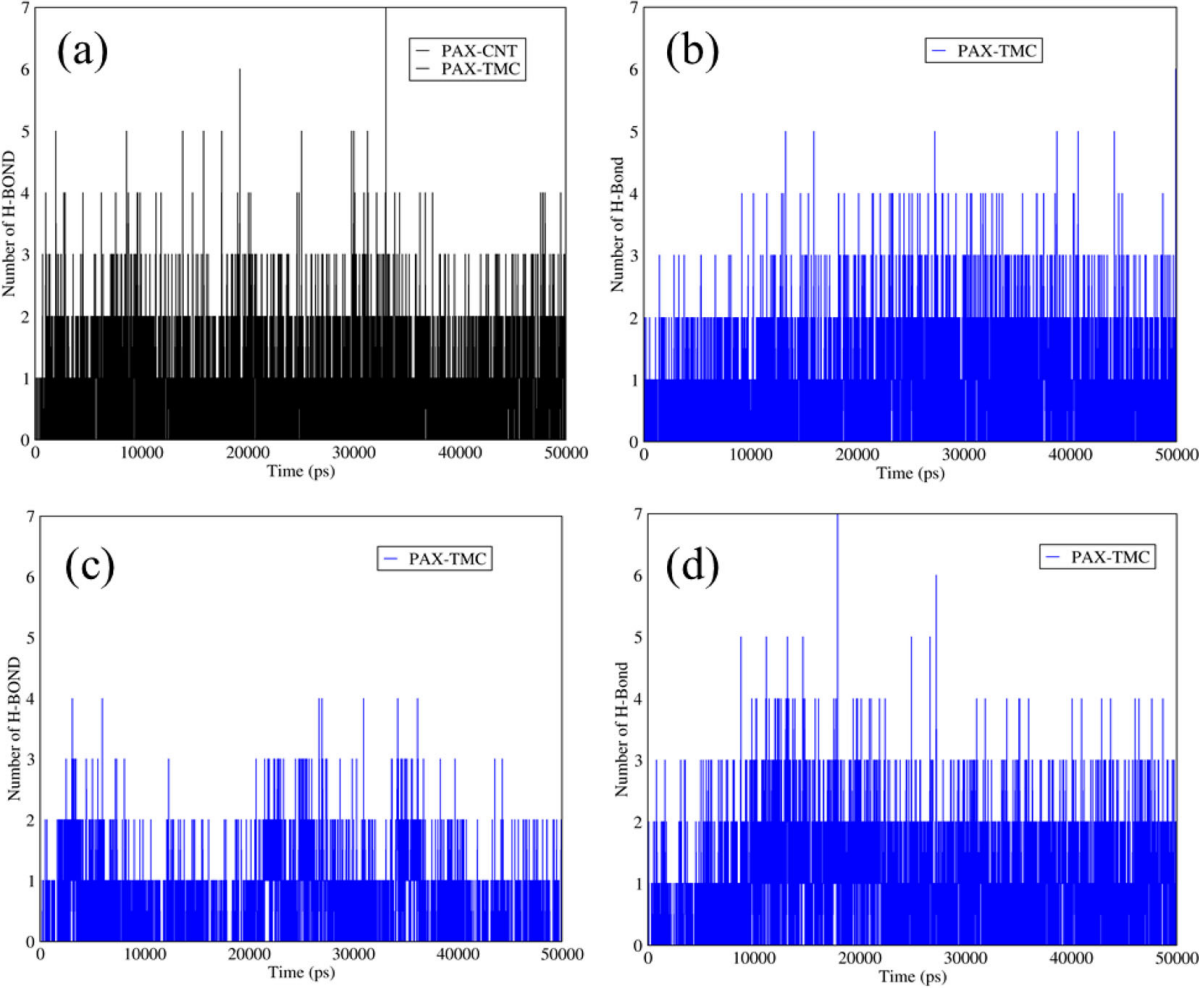
The number of hydrogen bonds between PAX and TMC in (**a**) PAX-TMC-SWCNTs and (**b**) PAX-TMC-graphene *versus* time at neutral pH; The number of hydrogen bonds between PAX and TMC in (**c**) PAX-TMC-SWCNTs and (**d**) PAX-TMC-graphene *versus* time at acidic pH.

Figure 9 demonstrates the number of hydrogen bonds between PAX and TMC in the structures of functionalized SWCNTs and graphene. As it is reflected in Fig. 9 (a, b), at neutral pH the average number of hydrogen bonds between the PAX and TMC in the mixtures of PAX-TMC-SWCNTs and PAX-TMC-graphene was around three, which is related to the polar structure of TMC that can provide the conditions for forming the hydrogen bonds. In addition, at the releasing pH, the number of hydrogen bonds between the TMC and PAX in these mixtures was approximately in the range of 2–3 (Fig. 9 (c, d)).

### Gyration Radii of DOX-TMC-SWCNTs and PAX-TMC-Graphene

**Fig. 10:**
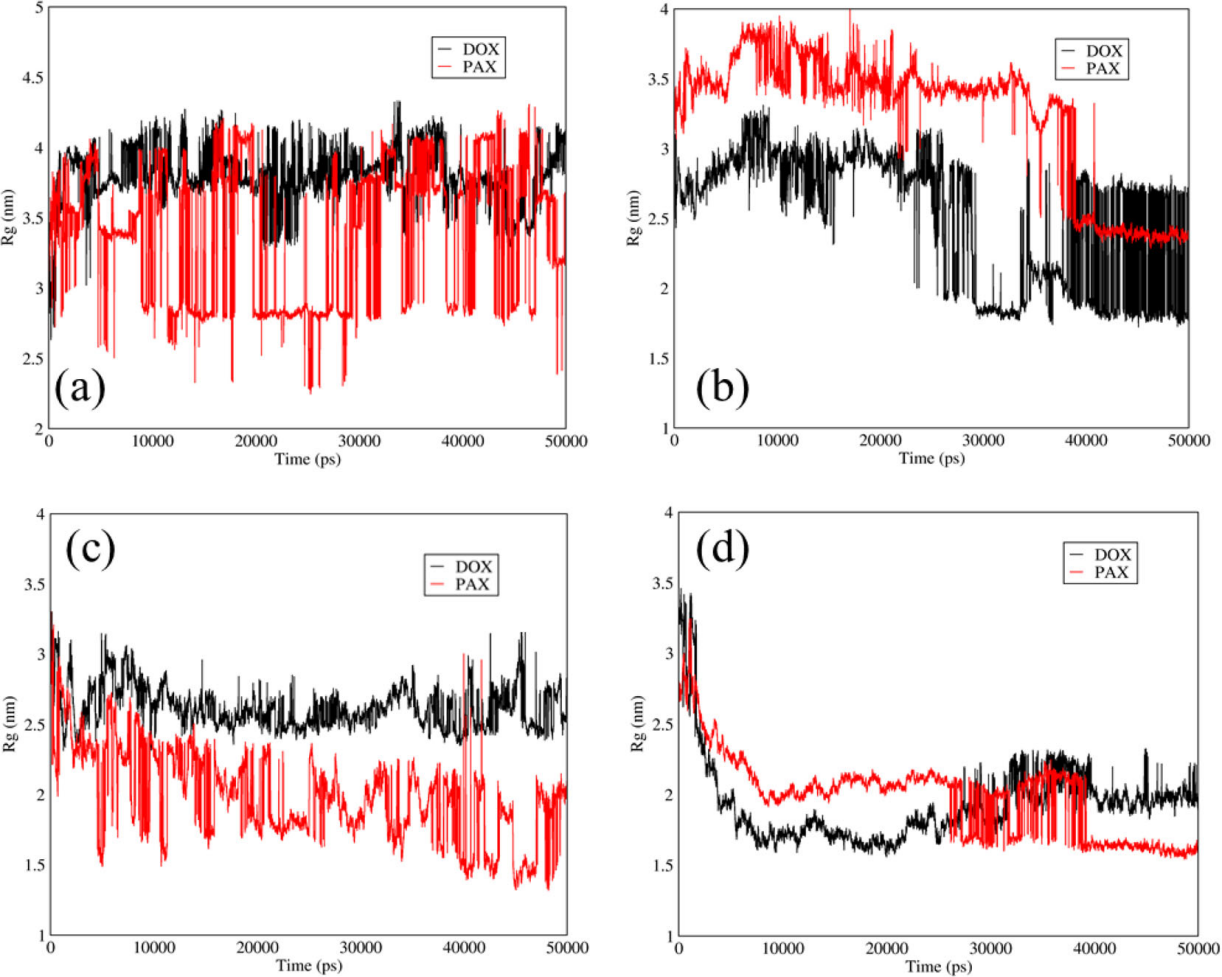
Gyration radii of DOX and PAX *versus* time at neutral and acidic pH for DOX and PAX in the mixtures of DOX-TMC-SWCNTs and PAX-TMC-graphene: (**a**) SWCNTs at neutral pH; (**b**) graphene at neutral pH; (**c**) SWCNTS at acidic pH, and (**d**) graphene at acidic pH.

Figure 10 indicates the gyration radii of the DOX and PAX in SWCNTs and graphene carriers at neutral and acidic pH. The gyration radius is a factor for evaluation of the size change of bio-macromolecules (such as proteins) and aggregation of the molecules (such as polymers). When the decrease of gyration radius is higher, the more stable carrier will be obtained and a more strong interaction between carriers and the drugs will be achieved. According to the relative curve of the PAX and DOX in Fig. 10 (a), the decrease of radius gyration of the PAX was higher than DOX and it shows that SWCNTs could be a more stable carrier for PAX at neutral pH. In addition, as can be seen in Fig. 10 (b) at neutral pH, the reduction of radius gyration for the PAX and graphene was higher than that of the DOX and graphene; so, it can be stated that the graphene can be a more stable carrier for the PAX. At the releasing pH, based on the relative curve of the PAX for both carriers, graphene was a more stable carrier (Fig. 10 (c, d)).

### Radial Distribution of DOX and PAX in TMC-SWCNTs and TMC-Graphene

**Fig. 11:**
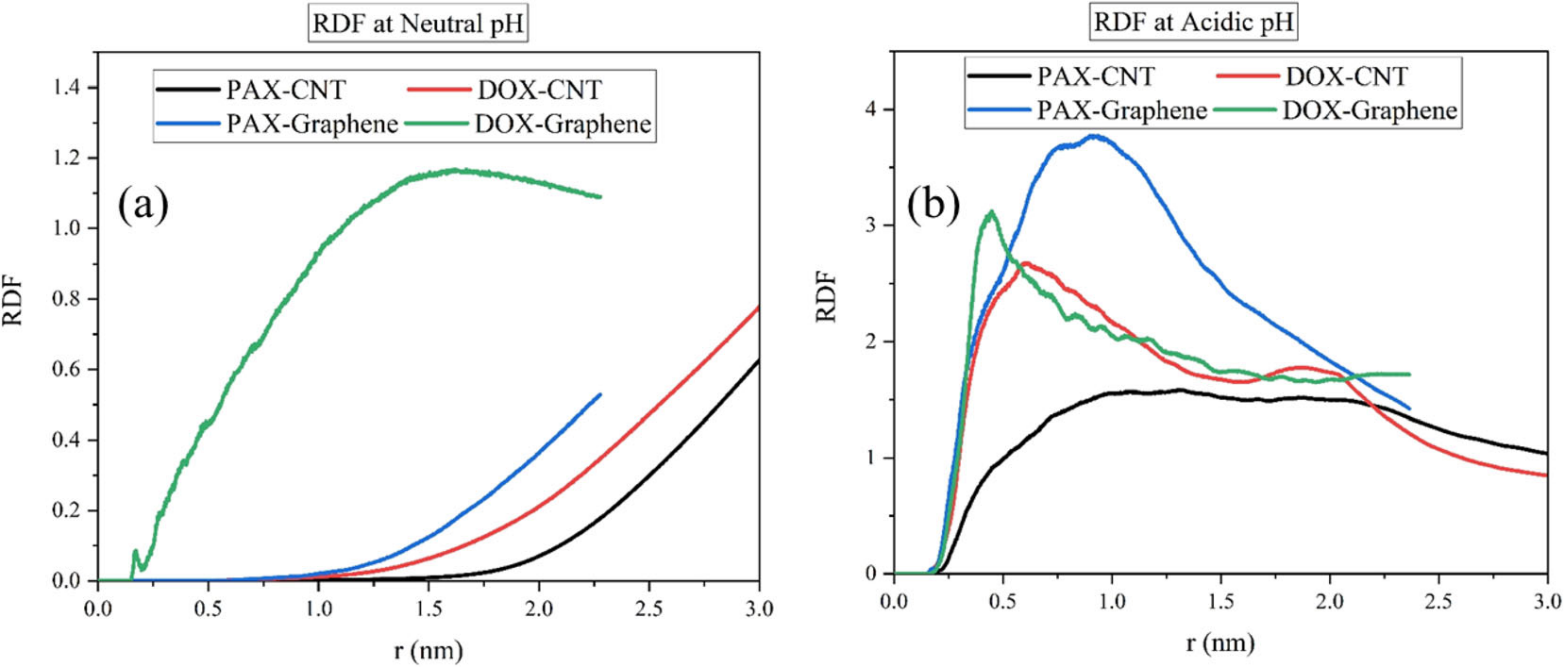
Radial distribution of the DOX and PAX in the mixtures of DOX-TMC-SWCNTs and PAX-TMC-graphene (**a**) at neutral pH and (**b**) at acidic pH.

Figure 11 exhibits the molecular dispersion of the DOX and PAX in the mixtures of TMC-SWCNTs and TMC-graphene at neutral and acidic pH, respectively. In this figure, a sharper curve is attributed to the concentration of drug molecules and the carrier at one point. As can be observed in Fig. 11, the DOX represented a higher radial distribution in comparison with the PAX in the mixtures of both carriers at both acidic and neutral pH. According to Fig. 11 (a, b), the DOX had a higher RDF value in the mixture of DOX-SWCNTs, which was about 0.8. The higher value of RDF can be related to better adsorption; thus, based on Fig. 11 (a) it is proved that DOX-SWCNTs had a stronger electrostatic interaction at blood pH and more proper adsorption could occur. At acidic pH, the DOX with both carriers led to a sharper curve and is understood that the molecules of the drug and carrier were of a higher concentration at one point. Thereafter, in the mixtures of DOX-graphene and DOX-SWCNTs, the DOX reflected a higher aggregation in comparison with the PAX. Furthermore, at the releasing condition, the maximum value of the RDF was around four, which belongs to the DOX-graphene, however, the PAX-SWCNTs had the minimum RDF value of approximately 1.5.

### Mean Square Displacement (MSD) of DOX-SWCNTs and PAX-SWCNTS

**Fig. 12:**
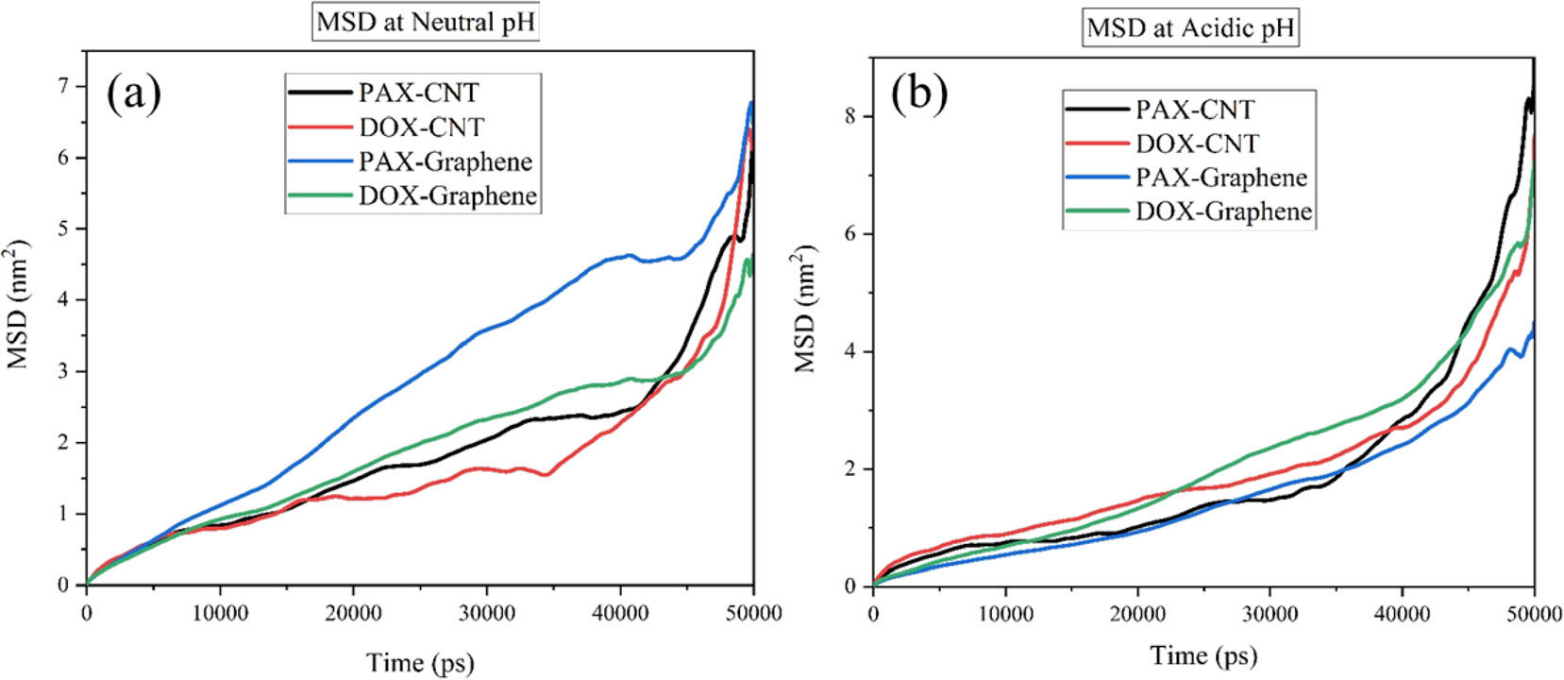
Mean Square Displacement of DOX in the mixtures of DOX-SWCNTs and PAX-graphene at **(a**) acidic pH and (**b**) neutral pH.

Mean Square Displacement (MSD) of DOX-SWCNTs and PAX-SWCNTS at neutral and acidic pH values are shown in Fig. 12. As it is clear in the figure, at neutral pH, the MSD was higher, which implies that the drug could be absorbed more quickly by the carrier; in contrast, the drug could be released from the carrier more slowly in the less MSD values. In fact, the longer half-life corresponds to the less MSD value, where the drug will be released from the carrier slower. In this figure, the slope of the graph shows the diffusion coefficient.

According to Fig. 12(a) at adsorption condition, at first all curves had a slight slope and by passing the time, the slope was increased. The enhancement of the slope has alluded to the spatial blockage between the molecules in which after some seconds, the spatial blockage was reduced and the rate of drug adsorption on these carriers could be altered. Among all cases in Fig. 12 (a), the PAX showed a significant adsorption behavior; however, the lowest adsorption rate as achieved for the DOX on the SWCNTs. Moreover, DOX showed a lower MSD value for release of PAX-graphene in releasing condition which means it can be released slower than DOX-SWCNTS, DOX-graphene and PAX-SWCNTS in the same condition. In addition, based on Fig. 11(b) the slopes of all curves were increased gradually, which can confirm that by passing the time, the spatial obstruction was reduced. Therefore, the ideal manner of drug adsorption and release belongs to the PAX-graphene, which is originated from the Van der Waals energy bonds between the PAX and Graphene.

## Conclusion

The side effects of anticancer drugs such as DOX and PAX will be reduced by developing biocompatibility and solubility of the drug through adding TMC to the mixture of DOX or PAX-SWCNTs or graphene and functionalizing the carriers by carboxyl group. In this MDs study, according to the electrostatic and Van der Waals energy results, at the blood pH, the DOX could be adsorbed by both functionalized SWCNTs and graphene and value of the electrostatic energy was around 6000 kj/mol and both carriers were ideal for the DOX loading. In addition, the electrostatic and Van der Waals energy between PAX and both carriers were around zero and there were no strong electrostatic and Van der Waals interactions between the PAX and these carriers at both neutral and acidic pH. Using TMC in addition to promoting the hydrophilicity and biocompatibility of these carriers, could facilitate the release of the DOX. Moreover, the TMC had a positive surface charge at acidic pH that was same as that of the amine group in DOX, which could facilitate the release of the drug at acidic condition. Furthermore, the number of hydrogen bonds between the drugs and carriers was increased by adding TMC at both acidic and neutral pH and adding TMC had a positive impact on promoting the formation of hydrogen bonds. As it was shown by gyration radius parameter, the highest dispersion at neutral pH was achieved for the PAX loaded on functionalized SWCNTs carrier while the DOX-graphene showed the highest concentration of the drug at a point. Overall, it can be stated that the PAX with functionalized graphene-TMC exhibited a more stable carrier in comparison with the DOX with the functionalized SWCNTs-TMC. Based on the MSD results, it was found that the PAX-graphene had the most proper drug adsorption and release behavior, where the PAX could be adsorbed quickly and be released slowly, leading to an increase in the drug’s half-life.
